# The Role of European Schools and University Departments of Public Health in the 2020 COVID-19 Response, European Region, 2020

**DOI:** 10.3389/ijph.2021.1604138

**Published:** 2021-10-07

**Authors:** Ariane Bauernfeind, Anders Foldspang, Alberto Fernandez-Ajuria, Robert Otok, John Middleton

**Affiliations:** ^1^ Andalusian School of Public Health, Granada, Spain; ^2^ Department of Public Health, Aarhus University, Aarhus, Denmark; ^3^ Association of Schools of Public Health in the European Region (ASPHER), Brussels, Belgium; ^4^ Faculty of Education, Health and Wellbeing, University of Wolverhampton, Wolverhampton, United Kingdom

**Keywords:** public health, COVID - 19, research, education, communication

## Abstract

**Objective:** The main objective was to examine, how European Schools of Public Health (SPHs) responded to the COVID-19 pandemic through 2020, across the main activity domains of the SPHs.

**Methods:** A cross-sectional survey based on an online questionnaire concerning the anti-COVID-19 activities from 1st March to 31st October 2020 of the 117 members of the Association of Schools of Public Health in the European Region (ASPHER). The questionnaire asked about 33 sub-themes within the four main themes of teaching, health communication to the public, research, and consultancy/advice.

**Results:** Fifty-nine SPHs (50%) completed the questionnaire. Seventy-nine per cent of participants were involved in COVID-19 related teaching; health communication to the public, 76%; research, 80%; consultancy/advice, 81%. Eight out of ten participants had been involved in all of the four main themes.

**Conclusion:** The study demonstrated a substantial body of COVID-19 related work by SPHs in Europe, and an outstanding potential to deliver crucial knowledge and skills to support the governance and the public health systems necessary to combat COVID-19.

## Introduction

The COVID-19 pandemic places the public health discipline at the centre of public attention. The pandemic is showing repeated waves of infection and a high death toll in countries all over the world. It threatens the health of individual citizens, higher-risk population groups and society as a whole. It threatens national and global economies.

It is a crucial and ethical requirement to activate all the available resources of public health knowledge and skills, nationally as well as internationally, in response to the pandemic. Universities, Schools and Departments of Public Health (SPH) have important resources in terms of competences. Research, continued education and training of professionals are key foundations for the implementation of a relevant, effective, ethically and economically acceptable intervention. Communications between schools are also necessary at various levels, for inspiration, peer support and developing consensus in policy and service responses.

The membership of the Association of Schools of Public Health in the European Region (ASPHER) is a substantial resource of public health knowledge, skills and expertise. ASPHER represents a diverse group of public health schools and research institutions, most of them being part of universities and often but not always of sections of medical schools and departments. They are of various sizes and act at various operational levels – national, regional and local. The number of ASPHER SPHs has increased substantially to now 117 full members from 42 European countries plus 10 associate members from other parts of the World [1, 2].

Continued education, training and research play central roles in public health, in services as well as academia [3]. Thus there is an accumulated body of knowledge of competencies developed by the membership of ASPHER over many years. Public health competencies comply with the main categories of the Essential Public Health Operations (EPHOs) as listed in Table 1 [3, 4].

**TABLE 1:** Anti-COVID-19 activities performed by European public health schools and departments of public health from 1st March to 31st October 2020, within teaching, health communication to the public, research and consultancy, by Essential Public Health Operation (EPHO) category. Fifty-nine members of the Association of Schools of Public Health in the European Region (ASPHER), 2020.

EPHO category; anti-COVID-19 theme	Teaching		Health communication to the public		Research		Consultancy/Advice		Activities
	N^a^	%^b^	N^a^	%^b^	N^a^	%^b^	N^a^	%^b^	N
**1. Surveillance of population health and wellbeing.**									
Surveillance	25	42	19	32	21	36	21	36	
Epidemiologic literacy	16	27	20	34	11	19	16	27	
Epidemiologic indicators for the management of the pandemic	29	49	28	47	21	36	29	49	
Applied/field epidemiology	14	24	11	19	9	15	11	19	
Outbreak investigation	20	34	11	19	10	17	12	20	
Prediction of epidemic development, mathematical modelling, patterns, comorbidities	18	31	17	29	18	31	15	25	
N of activities reported	122		106		90		104		422
**2. Monitoring and response to health hazards and emergencies.**									
Anti-epidemic strategy development, implementation and monitoring	16	27	20	34	15	25	22	37	
N of activities reported	16		20		15		22		73
**3. Health protection, including environmental and occupational health, food safety and others.**									
Infection prevention and control and preparedness for COVID-19 in healthcare settings and nursing homes, Personal Protective Equipment (PPE)	21	36	16	27	16	27	16	27	
Infection high-risk environments, e.g., nursing homes, schools, supermarkets, ballrooms, sports facilities, cultural facilities, other	14	24	17	29	16	27	17	29	
Occupational health	17	29	13	22	8	14	12	20	
Environment (climate, pollution)	10	17	4	7	7	12	4	7	
N of activities reported	62		50		47		49		208
**4. Health promotion, including action to address social determinants and health inequity.**									
Social determinants: infection or fatality high risk (vulnerable) population groups, e.g., elderly, other	23	39	15	25	15	25	14	24	
Health inequity	17	29	10	17	14	24	7	12	
Refugees and migrants	11	19	7	12	5	8	2	3	
Minorities and vulnerable groups	15	25	8	14	9	15	7	12	
Children’s health	9	15	5	8	7	12	5	8	
Impact on people with chronic conditions	16	27	8	14	11	19	9	15	
Mental health	18	31	15	25	23	39	11	19	
Social and individual behaviour, including interpersonal violence	14	24	6	10	10	17	6	10	
Voluntarism—motivation, contribution, management, impact	6	10	4	7	3	5	4	7	
N of activities reported	129		78		97		65		369
**5. Disease prevention including early detection of illness.**									
Testing theory, strategy, practice, validity and accuracy of test	10	17	8	14	9	15	11	19	
Prevention and infection control, confinement: methods, effects, ethics	25	42	25	42	17	29	30	51	
Vaccine (production, distribution, characteristics, equitable access)	12	20	13	22	5	8	12	20	
Contact tracing	15	25	15	25	8	14	18	31	
N of activities reported	62		61		39		71		233
**6. Assuring governance for health and wellbeing.**									
Health services organisation and management	24	41	13	22	19	32	17	29	
N of activities reported	24		13		19		17		73
**7. Assuring a sufficient and competent public health workforce.**									
Capacity of health services, the health workforce	15	25	7	12	14	24	12	20	
Peer to peer teaching, e.g., School of Patients	5	8	1	2	1	2	1	2	
N of activities reported	20		8		15		13		56
**8. Assuring organizational structures and financing.**									
Health economics of the pandemic, socio-economic impact, cost-effectiveness of interventions	14	24	5	8	7	12	7	12	
N of activities reported	14		5		7		7		33
**9. Advocacy, communication and social mobilization for health.**									
Advocacy	6	10	9	15	3	5	2	3	
Health communication, health literacy	20	34	14	24	11	19	11	19	
N of activities reported	26		23		14		13		76
**10. Advancing public health research to inform policy and practice.**									
Data management and data analysis	16	27	9	15	11	19	7	12	
N of activities reported	16		9		11		7		43

a Number of respondent schools reporting the anti-COVID.

b Percentage among respondent schools (N = 59).

In order that public health professionals will hold the competency profile necessary to deliver the EPHOs, ASPHER, partly together with the World Health Organization (WHO), has developed competency systems and lists for the public health workforce [5] as well as for the academic knowledge and skills foundation of public health [6]. These competencies apply to individual public health professionals as well as to public health institutions and systems [7]. A pilot survey among SPHs in four European countries showed that the schools generally covered the main components of competences and EPHOs [8], and SPHs can become local public health centres with regional, national and international potentials [9]. Moreover, the range of skills and expertise needed to combat modern global health concerns has expanded, requiring partnerships with a still widening range of disciplines, in natural and social sciences as well as humanities [10], and the formation of coherent networks of SPHs has for long been a natural matter of consideration [11].

ASPHER believes that SPHs are playing a critical role now but also will do so in the aftermath of the acute phases of the COVID-19 pandemic [12]. ASPHER performed this survey to throw light on the role of its members in the pandemic. The survey is one of an extensive range of activities by its COVID-19 Task Force [13]. ASPHER’s institutional members’ anti-COVID-19 activity range from specific training to reinforcing health communication to the public, producing and disseminating evidence, and providing advice to political and administrative bodies. Mapping ideas and best practices may stimulate and help design the role of SPHs beyond the present COVID-19 situation as well as help effectively combat the present as well as future pandemics. Mapping activities should be a source of inspiration and mutual support for SPHs.

The main objective of the present study thus was to examine, how European SPHs responded concretely to the challenge of the COVID-19 pandemic through 2020, within teaching, health communication to the public, research, and consultancy/advice.

## Methods

We performed a cross-sectional survey of the anti-COVID-19 activities of ASPHER’s member SPHs. Activities were concretely defined as responses carried out by SPHs, listed in the questionnaire, from teaching, health communication to the public, research to consultancy/advice with detailed 33 subthemes. The survey covered the period from 1st March to 31st October 2020. The target population of the study was all ASPHER affiliated SPHs, including 117 full members. Full members are defined as “Schools/teaching institutions, scientific/research institutes, and other structures with a role in education and/or training in public health, established within the European Region as defined by the World Health Organization [14, 15].”

Firstly, we piloted the survey to gain information to improve the efficiency of the main survey and to test the questionnaire [16]. Resulting adjustments were implemented in the main survey.

The data collection of the survey was performed from week 48, 2020, to week 2, 2021. Each institution received one token coded link. We used an online questionnaire, LimeSurvey^®^, a free and open-source statistical survey web app, which may be used with different web browsers and equipment (computers, tablets and cell phones). Respondents could move forward and backwards in the online questionnaire. As an extra tool for completing the web-based questionnaire, a paper version was offered to non-respondents, who complained of challenges to identify the existence of all questionnaire components in their institution.

Most of the questions were closed with a few open text questions. Participants were able to select multiple responses or to skip a list if they did not have any of the required information. To achieve relatively high sensitivity of the questionnaire tool, i.e., to miss as little as possible, we developed relatively detailed items in the repeated list combined with an open answer possibility. Respondents were asked about the same 33 sub-themes in all of the four main themes: teaching, health communication to the public, research and consultancy/advice. The four main themes with their 33 sub-themes were then grouped under the ten Essential Public Health Operation (EPHO) main categories.

We did not present any specific hypotheses regarding, e.g., the association between activities and types and size of institution, and we did not perform any statistical analysis. The report simply presents all answers to the full questionnaire and expresses findings in total counts and percentages among responding schools. The basis for the table is all respondents (N = 59). Percentages have been rounded and thus may not add to 100%.

## Results

Fifty-nine (50%) out of 117 full ASPHER member SPHs responded to the survey, reflecting 32 countries from a total of 42 ASPHER member countries. They are situated in 32 out of the 53 WHO European member states. The non-responding schools are located in ten more countries of the WHO European Region. Basic characteristics of the organisational structure of the individual SPH, such as private/public institution, university department, school of public health, did not vary significantly between respondents and non-respondents.

Seventy-nine per cent of participants were involved in COVID-19 related education or training activities; 76% of the institutions communicated to the public on COVID-19 issues; 80% were involved in research related to the COVID-19 pandemic; 81% had advised public authorities within public health, health administration, university education or governments at national, regional or local levels. Eight out of ten participants had been involved in all four main categories (Table 1).

The 33 sub-themes were grouped according to EPHO main category. The largest number of subtheme activities reported (422 subtheme activities) was under EPHO 1 (surveillance of population health and wellbeing), followed by EPHO 4 (health promotion, including action to address social determinants and health inequity, 369 activities), and EPHO 5 (disease prevention including early detection of illness, 233 activities) (Table 1).

### Teaching

Fifty schools/departments were involved in COVID-19 related education or training activities. The main COVID-19 themes taught to public health students (bachelor, master, PhD) were “epidemiologic indicators for the management of the pandemic” (49%), followed by “surveillance and prevention and infection control” (42%). “Health service organisation and social determinants” were not less important and taught at 41 and 39% of respondent SPHs, respectively. Additional themes listed in free-text comments were crisis management, risk communication, public health law, and social anxiety.

Themes taught to social workers, psychologists, nurses, midwives, carers, and other health personnel, were dominated by “Infection prevention and control” (29%) and “Infection prevention and control and preparedness for COVID-19 in healthcare settings and nursing homes” (29%). With almost the same frequency: “epidemiologic literacy” (27%) and “epidemiologic indicators for the management of the pandemic” and “surveillance” (24%). Additional themes listed were hygiene (disinfection), quality of health services, and the use of digital solutions for outbreak investigation.

As concerns teaching methods, forty-four institutions used “distance training” strategies, 24 “written materials,” and 23 “blended/hybrid” learning for their public health students. Among additional teaching methods, internship at disease control centres, practical training for contact tracing, role-play, serious games, and film making were listed.

For the group other than public health students, “distance learning” was applied in twenty-six institutions, while 15 used “written material” and 11 “social media.” “Classroom teaching (max. 20 students)” was less used (9 institutions) in this target group, as compared to 17 institutions for public health students.

### Health Communication to the Public

Almost half (47%) of the institutions communicated to the public about “epidemiologic indicators for the management of the pandemic,” followed by “prevention and infection control, confinement: methods, effects, ethics” (42%), “epidemiologic literacy” and “anti-epidemic strategy development, implementation and monitoring” (both 33%). The least communicated themes were about the “environment” (7%), “voluntarism” (7%), and “peer to peer teaching” (2%). Besides, respondents in the free-text reported having communicated to the public about breaking myths about 5G and COVID-19 relations; lifestyle changes during the outbreak; ventilation; indoor air; surface cleaning; group gatherings; practices.

Thirty-five respondents reported having used “interviews on radio and TV,” “interviews in newspapers and periodicals,” and “social media,” to communicate COVID-19 themes to the public.

### Research

A significant range of themes was covered in the schools’ research participation and scientific publications. The two most frequently reported themes were “mental health” (39%), “surveillance” and “epidemiologic indicators for the management of the pandemic” (both 36%).

Other research themes were:

• Seroprevalence studies performed in the general population.
• Serological studies.
• Community health: young and adolescents and COVID-19.
• Drug research against COVID-19.
• Health behaviours and social distancing during lock-down.
• Lifestyle changes during the outbreak, e.g., changes in alcohol consumption during the COVID-19 pandemic.
• Digital health literacy.
• Bodyweight during the COVID-19 quarantine.
• Medical ethics.

A quarter of respondents had published about “epidemiologic indicators for the management of the pandemic” (27%) and “surveillance” (24%), followed by “prevention and infection control, confinement: methods, effects, ethics” (22%) and “health services organisation and management” (20%).

Seventeen out of fifty-nine participants reported that their COVID-19 related research had an impact on political decisions by:

• Defining national guidelines in COVID-19 infection control and prevention.
• Feeding into the anti-epidemic strategy of the country.
• Informing stakeholders of the levels of community spread of COVID-19.

### Consultancy/Advice

A wide range of themes of advice was given to public health authorities. More than half of the institutions had advised about “prevention and infection control, confinement: methods, effects, ethics” (51%), “epidemiologic indicators for the management of the pandemic” (49%), and “surveillance” (44%).

More than a third of respondents reported that their advice had an impact on political decisions, described in free text as:

• Defining national policies.
• Deciding on anti-epidemic strategy development, implementation and monitoring, infection control and testing strategy, quarantine and closure of the country.
• Shaping policy decisions concerning contact tracing, openings/closings of businesses, mask-wearing, school openings/closings, population-based testing.
• Designing temporary COVID-19 hospitals.

In relation to COVID-19, more than half (34 out of 59) responded that they had been directly involved with public authorities at various decision levels, namely the “national parliament,” the “Ministry of Health,” the “national advisory expert committee,” the “national public health council,” “regional task forces,” “regional health authorities,” the “armed forces,” “local health authorities,” and “hospital level.”

## Discussion

Our findings show an impressive engagement of Schools and University Departments of Public Health (SPHs) in the COVID-19 pandemic in teaching, health communication to the public, research, and involvement in the work of central decision-making bodies by providing consultancy/advice.

About half of ASPHER member schools and departments participated in the survey. The findings must be considered with caution if taken as a full and valid representation of the anti-COVID-19 combat profile of ASPHER member SPHs. The findings, however, represent a minimum of overall anti-COVID-19 activity by SPHs in the WHO European Region. The findings appear sufficient to answer the main questions of this research, namely whether SPHs have invested their knowledge and skills in combatting the pandemic during 2020, and whether the invested competences represent very selected areas or a broad range of public health resources. The last has been shown here to be the case, so that eight out of ten schools demonstrated involvement in all four sections of the questionnaire: teaching, health communication, research and consultancy/advice, with major weight on themes instrumental to the central aspects of the pandemic’s development. Moreover, the whole spectrum of EPHO main categories was represented. This is in balance with the findings of Bjegovic-Mikanovic et al. [17] when they about 10 years ago documented large numbers of public health components and aspects delivered by SPHs in the European Region.

It is likely that the results presented are a minimum level of anti-COVID-19 activity. Some respondents reported difficulties gathering all the anti-COVID-19 activity in their institutions, which may have resulted in under-reporting. The survey explored COVID-19 related teaching, health communication to the public, research, and consultancy/advice, performed or planned during the March to October 2020 period. Such activities were specific activities often or mostly added to the standard on-going activities, which were also affected by the pandemic. The special activity patterns in these schools and departments indicate the effort and commitment to combatting the pandemic. We did not explore the ability and concrete planning to continue the reported activities or start other anti-COVID-19 activities. In addition to delivering anti-COVID-19 efforts of high quality, the schools struggled to adapt their regular activities to repeated demands to close and re-open [18].

The willingness to invest resources in the 2020 anti-COVID-19 combat was demonstrated already in teaching, where many opened up to a wider audience of students to answer the demand of nursing homes and primary health care. Flexibility was also seen in the offer of themes covering “infection control,” “personal protection” and “health care management,” besides more classical public health themes like “surveillance” and “field epidemiology.”

Given the confinement situation in most of the countries during the time period covered, teaching was mainly done through “distance” and “blended/hybrid” methods. However, some institutions did not limit themselves to that and introduced challenges like an “internship at disease control centres,” “serious games,” “film making” and “role-play” - to list some.

SPHs showed a pro-active approach to health communication to the public through social media and interviews, mainly concerning “epidemiologic indicators” for the “management of the pandemic, and prevention and infection control.”

Eight out of ten participants had been involved in anti-COVID-19 research. The range of research themes covered was wide, dominated by “surveillance,” “epidemiologic indicators,” “prevention and infection control,” and “mental health.” The evidence produced informed national guidelines, national strategies, and political decision making.

Advice and consultancy were also strongly covered, mainly in “prevention and infection control,” “epidemiologic indicators for the management of the pandemic,” “mathematical pandemic prediction models,” and more general “surveillance.” Importantly, the engagement was at high decision levels. Some institutions took direct part in national task forces, while others advised their Ministry of Health at national and also at local levels. Advice was given to and through schools, universities, workplaces and churches. A range of professional networks represented other communication channels. The advice given defined national policies and strategies concerning, e.g., decisions on contact tracing, confinement, mask-wearing, and closing of schools.

The main impression from the survey is that of the unveiling of existing anti-COVID-19 combat relevant resources. University cultures, with their basic principle of freedom of research, are only to some extent considered possible parts of society’s public health standard procedures or tools. Here we have seen them step up to the challenge and play a central and important role. The study shows the availability of these resources to join the emergency response to situations, which are catastrophic for the population’s health, like the present one. The study also demonstrates the need to enhance and grow public health expertise, investing in programmes of teaching and training to create the next generation of public health professionals.

In many European countries, a coherent organisation of comprehensive public health is to a large degree lacking. “Health services” are “disease services,” and prevention of ill-health and health improvement lag behind the coherence of the organisation of medical curative systems [19]. Years of austerity policies in many countries have taken an additional toll in terms of poor health, including increased mortality, in some nations and some population sectors. The pandemic has shown up grotesque inequalities in health, within and between countries [20]. The impacts of the virus and the impacts of lockdowns have fallen unequally on minorities, those in high-risk occupations and the poor, already living with vulnerability to ill health and premature death [21, 22].

The biomedical sides of public health have often been over-emphasised at the expense of indispensable social theories and action, necessary in, for instance, societal lockdowns and contact tracing in pandemics like the present one [23]. The development of rationally goal-oriented, comprehensive and coherent public health policies and strategies takes time, not least because they must be based on social, social-psychological behavioural, and health economic dimensions besides the biomedical components [24].

We have seen a poor state of preparedness in many countries in Europe, neglect of pandemic planning and deliberate disinvestment in public health resources [25, 26].

We have also seen the politicisation of public health science, to disastrous effect in many European countries [27]. The next generation of public health professionals will need to be politically astute and alive to the potential of social media and digital technologies to improve or damage the health of the public [28]. Our public health professionals will need to be strong on analytic competencies and assessment of the quality of scientific and practical evidence, leadership, knowledgeable on health economics and the law and building their actions on a strong ethical framework. They will also need to be recognised for their expertise, professionalism and authority [29].

The present study has demonstrated that the necessary components do exist for creating a resilient, expert and comprehensive public health system, in many European countries. They are here, but they do not exist everywhere or at equal quality levels, in all countries or in all university environments and SPHs. There is a challenge and an imperative for countries to work together to enhance our public health systems and preparedness for the future [30–32]. In all this, education and training, interacting with practice and research, are central requirements.

Having examined the role of Schools and University Departments of Public Health in combatting the COVID-19 pandemic we can conclude that the involvement and roles taken are important and demonstrated impact. The SPHs have shown that they are able to deliver knowledge and skills, all together at a large scale and not exclusively for academic purposes but instrumental to practical public health analysis, planning, service intervention, implementation and evaluation, and without letting down their inborn free research obligation – as in the present pandemic. Activities included, on one end, very much down-to-earth procedures such as infection control and contact tracing, and, at the other end, a wide range of governance and policy advice and research.

This study is a milestone in the general development of theoretical and practical inputs to European public health services by European Schools and University Departments of Public Health. Moreover, it demonstrates an outstanding potential to yield concrete here-and-now support to the governance systems and the public health systems responsible for combatting COVID-19.

We recommend that these results are disseminated widely in order to increase celebrating the role of SPHs in combatting the ongoing COVID-19 pandemic and to serve as inspirational knowledge exchange.

Governments and international bodies must learn from the pandemic and apply economic, social and health policies which improve and protect health fairly and equally. They must build capacity for public health preparedness and response to epidemics—be they infectious or non-communicable. And they must acknowledge and recognise professionalism and expertise in public health. ASPHER members stand ready to support national governments and international agencies in meeting these aims. We must plan for an outbreak of health.
